# Perceived HRM and turnover intentions of elderly care workers: perspective from person-job fit and institutional ownership

**DOI:** 10.1186/s12912-024-01926-9

**Published:** 2024-04-15

**Authors:** Fang Wu, Wei Chen, Ruyi Wan, Jiatong Lu, Qianqian Yu, Qilei Tu

**Affiliations:** 1 School of International Pharmaceutical Business, China Pharmaceutical University, 639 Longmian Avenue, Nanjing, Jiangsu 211198 China; 2 Beijing College of Social Administration, No.2 Yanling Rd, East Yanjiao Development Zone, Beijing, 101601 China

**Keywords:** Elderly care workers, Perceived human resource management, Turnover intentions, Person-job fit, Institutional ownership

## Abstract

**Background:**

Although the phenomenon of high turnover rate in the elderly care industry has existed for a long time, there are few studies that have constructed frameworks to comprehensively analyze the strength of the effects of various factors on the turnover intention of elderly care workers.. This study analyzed the impact of different types of perceived human resource management practices on elderly care workers’ turnover intentions and whether this relationship is moderated by person-job fit and ownership of elderly care institutions.

**Methods:**

This is a cross-sectional and regional survey study. The study included questionnaire survey data from a total of 305 elderly care workers from 42 elderly care institutions in 21 provinces in China during June to September 2021. Descriptive statistics, Pearson correlation coefficient, multiple regression, and heterogeneity analyses were performed.

**Results:**

Perceived work environment ($\beta$=-0.5164, *p*< 0.01), perceived occupational protection ($\beta$=-0.3390, *p*< 0.01), perceived welfare benefits ($\beta$= -0.2620, *p*< 0.01) and perceived competency training ($\beta$= -0.1421, *p*< 0.1) were all significantly and negatively related to turnover intentions, the quality of perceived work environment has the greatest impact on elderly care workers’ turnover intentions. Under the moderating effects of person-job fit and ownership of elderly care institutions, there existed heterogeneity between perceived human resource management and turnover intentions among elderly care workers. High level of person-job fit and elderly care institutions’ public feature can effectively weaken the negative impact of each type of perceived human resource management on turnover intention among elderly care workers.

**Conclusions:**

The managers of elderly care institutions should optimize the management mechanism, typically pay attention to elderly care workers’ working environment, formulate and improve the professional standards and job requirements for elderly care workers, promote the public welfare value of nursing care services, and strengthen the sense of honor and responsibility of elderly care workers to reduce the turnover rate.

**Supplementary Information:**

The online version contains supplementary material available at 10.1186/s12912-024-01926-9.

## Introduction

Since the United Nations published The Aging of Populations and its Economic and Social Implications [1], how to meet the care needs of elderly has become a new topic of livelihood security in various countries [2]. Elderly care workers are an important source of high-quality elderly care services [3]. Compared to hospital nurses, the role of elderly care workers tends to focus on caring for the personal living and basic health care of the elderly. Although they do not have qualifications for medical skills such as injections and medication [4], they are an important force in alleviating the work pressure of medical teams and improving the efficiency of medical service supply [5]. However, currently the world is generally facing a high turnover rate of elderly care workers [6], and the World Health Organization predicted that the global turnover rate of elderly care workers would be as high as 40% in 2023 [7]. Under the background of population aging, the high occupational mobility of elderly care workers is not only detrimental to guarantee the quality of life of the elderly population, but also poses challenges to the health care systems of each country.

China entered aging population society in 1999 [8]. Between 2014 and 2020, the number of officially registered elderly care workers in China increased from 194,100 to 378,900 [9]. However, the high turnover rate of China’s elderly care workers has led to a workforce gap of at least 5 million in the elderly care industry due to several reasons such as low wages [10]. Currently, each elderly care worker in China cares for an average of six elderly people every day, which is higher than the general standard of 4:1 in developed countries [11]. The reason for this phenomenon is that social prejudice against elderly and the elderly care industry affects employees’ career choices, resulting in high turnover rate in the elderly care industry [12]. Therefore, reducing the willingness of elderly care workers to turnover has become an urgent issue in the process of building China’s elderly care service system.

At present, the issue of elderly care workers’ turnover problem has gradually become a hot topic of academic attention. A research review summarized the triggering factors of turnover intention among elderly care workers into physical, cognitive, and organizational three levels [13]. Among them, at physical level, the characteristics of the elderly care industry bring high-intensity physical pressure, which has become the main reason for the frequent turnover of elderly care workers [14], due to heavy work burden and incomplete mastery of nursing professional skills, elderly caregivers often suffer from pain in lower back, neck, and shoulder joints [15], feeling physically overwhelmed and choosing to resign; At cognitive level, some studies have pointed out that elderly care workers often fails to meet their personal values and career expectations [16, 17], low income, workplace violence, and low social status lead to occupational burnout among elderly care workers [18], they choose to resign due to lack of work motivation; In addition, factors at organizational level that affect the willingness of elderly care workers to resign are often believed to be several shortcomings in management of elderly care institutions, such as the failure to provide good work facilities and conditions [19], or unreasonable salary, benefits, and career promotion systems for employees [20], which results in the loss of elderly care workers.

Social information processing theory pointed out that employees extract information from the environmental background of organizational human resource management practices, spontaneously forming their own perceptions of human resource practices [21]. They are employees’ subjective perceptions of organizational human resource management, including direct perceptions of human resource management practices and evaluations of management practice intention [22]. Perceived human resource management have been proven to be moderated by elements such as job requirements, organizational structure [23], and can influence the sense of work experience [24], this sense of experience affects personal value judgments and mediates employee behavioral responses, and are a valid predictor of employee work behaviors [25, 26]. Turnover intention can be seen as a psychological response of employees after experiencing negative work experiences. Several studies have confirmed that employees’ perceived human resource management can significantly mediate occupational burnout and turnover behavior [27, 28], which is a strategic factor for the industry to cope with shocks, ensure talent supply, and improve organizational efficiency [29]. In the past decade, Chinese elderly care workers have had low levels of education and poor nursing skills [30], the expansion of elderly care worker team has been constrained by human resource management factors such as work environment, salary and benefits, and employment training for a long time. Therefore, it is necessary to systematically examine the impact of perceived human resource management of elderly care workers on their turnover intentions and the internal mechanisms among them. Solving these issues is beneficial for meeting the goal of China’s “The Medium-and Long-term Plan for Responding Proactively to Population Aging”, and help to reduce the turnover rate of elderly care workers.

## Research framework

### Relationship between perceived HRM and turnover intentions

The concept of turnover intention is affiliated with the individual level, employees’ turnover intentions have been proven to come from the results of evaluation of organizational human resource management practices [31]. The connotation of human resource management practice is broad, studying the relationship between employees’ perceived human resource management and turnover intentions from an individual perspective need to consider the impact of different aspects of employee’s perceived human resource practice, including evaluation of working conditions and compensation packages, as well as expectations of job security and career advancement [26]. In Herzberg’s *The motivation to work,* organization’s, human resource management means were classified as two dimensions of motivation and hygiene [32]. This study takes into account the unique characteristics of the elderly care industry and the availability of data, using Herzberg’s classification method to select work environment and occupational protection as two typical elements of “hygiene perceptions”, and set welfare benefits and competency training as two typical elements of “motivation perceptions” of elderly care workers.

*Hypothesis 1*: The perceived HRM of elderly care workers is significantly negatively correlated with their turnover intentions.

### Moderating role of person-job fit

The degree of person-job fit is an important perspective for predicting employee behavior. Person-job fit means that employees have sufficient knowledge and skills to meet the job requirements [33]. At a high level of person-job fit, employees have a good sense of self-efficacy due to a high degree of fit between their personal abilities and job requirements. Negative perceptions of human resource management practices were weaken under the moderation of employees’ self-confidence and sense of responsibility, thus reducing the risk of employees’ turnover intentions [34]; while at a low level of person-job fit , job errors due to personal incompetence, on the other hand, may be explained by the shortcoming of organizational human resource management practices driven by employees’ responsibility avoidance motives [35], and unreasonable human resource management practices reduce the sense of work experience and invite turnover behavior [36].

*Hypothesis 2*: Person-Job fit has a moderating effect between perceived HRM and turnover intentions.

### Moderating role of institutional ownership

Institutional ownership determines the management model and operational logic, and differences in working conditions across institutional ownership structures influence employees’ attitudes and behavioral decisions [37]. Chen et al. explained the reason for behavioral differences between public and private institutions’ employees from the perspective of intrinsic motivation, which was that the small operating system of public institutions meant that they cannot provide sufficient material incentives with limited resources [38]. The core function of serving the community had limited to attract job seekers seeking extrinsic incentives. Compared to the private institutions, employees in public institutions had stronger aspirations for job stability [39], suggesting that they were driven by public values and job security to overcome poor work experiences due to factors such as institutional management systems with higher levels of public service motivation [40], which externally manifested as lower turnover intentions. Therefore, this study attempts to prove the influence of the perceived human resource management practice of elderly care workers on turnover intentions, further evaluating the moderating role of person-job fit and institutional ownership.

*Hypothesis 3*: Institutional ownership has a moderating effect between perceived HRM and turnover intentions.

**Fig. 1:**
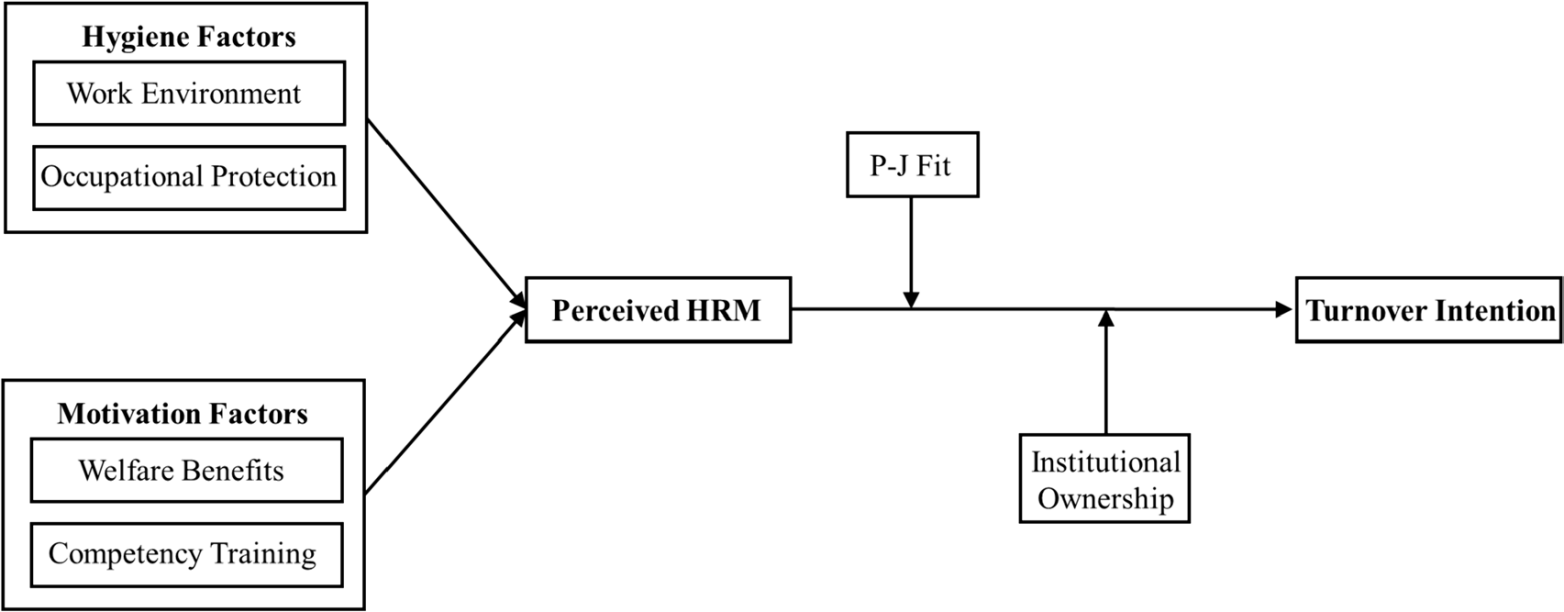
Research framework

The research framework of this paper is shown in Fig. 1.

## Methods

### Study design

The data used in this study were obtained from a questionnaire survey of carers for the elderly in China conducted from June to September 2021. Before conducting the survey, the researcher randomly selected some elderly care institutions in Nanjing, Jiangsu Province, and conducted semi-structured interviews with their managers. They have both a good understanding of the current status of the development of the team of local elderly care workers and a high level of awareness of the occupational mobility of the national elderly care workers. The design of the research questionnaire based on the collated interview results effectively ensured the credibility of the questionnaire design.

### Participants

**Fig. 2:**
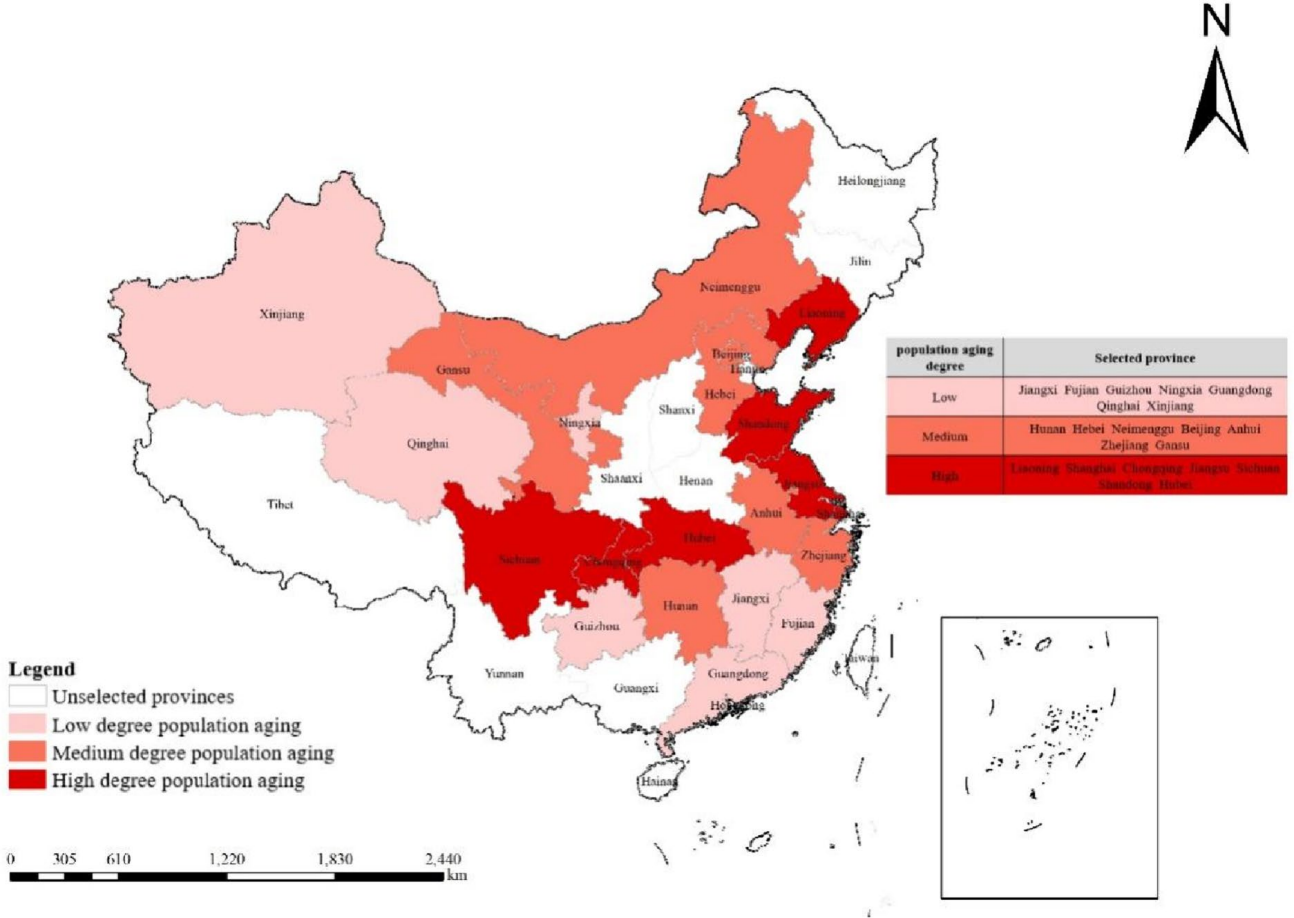
Twenty one selected sample research provinces

During formal survey, the research team divided 31 provinces in Chinese Mainland into 3 categories of high population aging, medium population aging and low population based on the principle of stratified sampling and the proportion of elderly people over 60 years old^1^, and then respectively selected 7 provinces of 3 categories. Due to the fact that elderly care workers in elderly care institutions are divided into three groups based on the self-care ability of the elderly people, which is “severe disability group”, “mild disability group”, and “complete self-care group”, therefore, in each elderly care institution, we follow the principle of stratified sampling and randomly select 4-5 elderly care workers from each group to fill out questionnaires. The provincial sampling results are shown in Fig. 2. Two elderly care institutions were randomly selected from each of the 21 provinces for face-to-face questionnaire data collection. After leading elderly care workers to become familiar with questionnaire content and filling out requirements, research team conducted face-to-face questionnaire data collection on elderly care workers one by one.

The questionnaire survey obtained informed consent from elderly care workers, ensuring anonymity and confidentiality throughout the process. Research team emphasized that there is no right or wrong answer to all questions, and the survey results are only for academic research purposes. A total of 527 questionnaires were distributed in the survey, 422 questionnaires were actually collected, with a recovery rate of 80.1%. Invalid questionnaires with missing values, overlapping contents, and inconsistent answers were excluded, and 305 valid questionnaires were finally obtained, with an effective rate of 72.3%.

### Ethical considerations

The Institute of Science and Technology of China Pharmaceutical University, as the Ethic Approval Committee approved the study. Since this study did not conduct human clinical or animal experiments, we only investigated the current work status of elderly care workers in institutions. The participants were informed about the purpose and procedures of this study. Informed consent was obtained from the elderly care workers for the questionnaire and confidentiality was assured throughout the survey.

### Variables and measures

A total of 21 items were compiled in the questionnaire^2^, including 6 items on turnover intention and Perceived HRM, 2 items on Person-job fit and institutional ownership, 7 items on individual characteristics such as age and gender, and 6 items on work status such as work burden and years.

#### Turnover intentions and perceived human resource management

On the one hand, to measure turnover intentions, we used the question, “How willing are you to turnover at this time?” The answer is based on a five-point Likert scale, including 1, very strong; 2, relatively strong; 3, average; 4, occasionally want to turnover; 5, will not turnover. To increase the reliability of the measurement of dependent variable, we designed another question, “Are you still willing to work in elderly care services in the future”, which can to some extent supplement the adverse effects caused by using only one question to measure dependent variable. On the other hand, perceived human resource management practices include both hygiene and motivation dimensions. We asked elderly care workers in the questionnaire, “Do you think the current working environment/occupational protection/welfare benefits/competency training of the institution is perfect?” The answers ranged from 1-5, indicating very satisfied, fairly satisfied, average, not very satisfied, and very dissatisfied. The Likert five-point scale was still used to score each of the four questions.

#### Person-job fit and institutional ownership

The two moderating factors that this paper focused on were person-job fit and institutional ownership. The question used to investigate person-job fit is: “How relevant is your previous study or work experience to your current job?” The five options were ranked by relevance:1, very relevant; 2, relatively relevant; 3, average; 4, not very relevant; 5, very irrelevant. Answers were scored on a five-point scale. On the other hand, we investigate whether the ownership of elderly care workers’ work units is public or private by asking: “What is the nature of your workplace?” Using binary variables of “0” and “1”.

#### Control variables

Based on existing studies [15, 41], control variables were considered to be selected in terms of both individual characteristics and work status of elderly care workers. Individual characteristics referred to the demographic information of elderly care workers, including the gender, age and education level of elderly care workers. Where the gender dummy variable took the value of “1” if the respondent’s gender was male, and “0” otherwise; age of elderly care workers was nominal age at the time of the survey; education level was a discrete variable with values ranging from 1 to 4, representing “primary school and below”, “junior high school, junior college or high school”, “college or university undergraduate”, and “master’s degree and above”, respectively. The factors considered in terms of working status included the number of elderly people served, years of experience in the field and the acquisition of vocational skills certificates. Among them, the number of elderly people served referred to the number of elderly people cared by elderly care workers for per day, with 1 indicating 0-10 people, 2 indicating 11-20 people, and 3 indicating 21-30 people; the options of years of experience in the field ranged from 1 to 5, indicating “5 years or less”, “(5, 10] years”, “(10, 15] years”, “(15, 20] years”, and “more than 20 years” respectively; the acquisition of vocational skill certificates was a dummy variable. If a carer for the elderly had already obtained an elderly care professional certificate, the value was “1”, otherwise it was “0”.

### Data analysis

Firstly, to test the overall reliability and validity of the questionnaire, reliability and validity tests are conducted on the questionnaire. The results indicate that the Cronbach’s $\alpha$ coefficient value for the 21 items in the questionnaire as a whole is 0.722, indicating acceptable reliability and overall stability of the questionnaire design; The overall KMO value of the questionnaire is 0.804, which passed the Bartlett’s spherical reading test (*p*<0.01), indicating that the questionnaire has good validity and can accurately measure research variables. Next, we conduct questionnaire data analysis, Firstly, descriptive statistics were used to calculate the frequencies, percentages, means, and standard deviations of individual characteristics and work status of elderly care workers,; Secondly, Pearson correlation analysis was used to initially calculate the correlation coefficients between the independent variable and dependent variable; Next, multiple regression analysis was used to verify the impact of perceived human resource management on elderly care workers’ turnover intentions; Finally we grouped the questionnaires to test the heterogeneous effects of person-job fit and institutional ownership.

### Model choosing

Although the dependent variable of this study, turnover intention, is a categorical variable and should be analyzed by ranking model, considering that the estimation results obtained by using OLS model are almost consistent with the Logit or Probit models provided that the regression model is set correctly [42]. To facilitate the verification of the moderating effects of person-job fit and institutional ownership, we choose linear regression model to examine the impact of perceived human resource management practice on turnover intention of elderly care workers.

## Results

### Sample description

Table 1 reports the results of descriptive statistics for personal characteristics and work status variables. Demographically, 78% of the 305 elderly care workers surveyed were female, with an average age below 40 years; in terms of work status, 73.77% of elderly care workers cared for <= 10 elderly people per day and 24.26% elderly care workers cared for (11, 20] elderly people per day, and they had a short average number of years of experience in the field with a certification rate of 70.16%.

**Table 1:** Descriptive statistics of personal characteristics and work status

**Variable**	**Category**	**Number**	**Percentage**	**Mean**	**SD**
Gender	Female	238	78.03%	0.2197	0.4147
	Male	67	21.97%		
Age	—	305	100%	37.7607	13.5539
Education	Primary	22	7.21%	2.4787	0.6338
	Middle	116	38.03%		
	college	166	54.43%		
	postgraduate	1	0.33%		
Elderly Number	0-10	225	73.77%	1.282	0.4925
	11-20	74	24.26%		
	21-30	6	1.97%		
Work Year	0-5	222	72.79%	1.4295	0.8862
	6-10	59	19.34%		
	11-15	10	3.28%		
	16-20	4	1.31%		
	Above 20	10	3.28%		
Certificate	Yes	214	70.16%	0.7016	0.4583
	No	91	29.84%		

### Correlations of study variables

The Pearson correlation coefficients of the core explanatory variables are shown in Table 2. The turnover intention is significantly and negatively related to perceived work environment ($r$=-0.377, *p*<0.01), perceived occupational protection ($r$=-0.2713, *p*<0.01), and perceived welfare benefits ($r$=-0.2318, *p*<0.01). There is no correlation between perceived competency training and turnover intentions, which is contrary to the previous hypothesis. The hypothesis needs to be further tested.

**Table 2:** Correlation coefficients of core explanatory variables (*n*=305)

	**Mean**	**SD**	**1**	**2**	**3**	**4**	**5**
1.Turnover Intentions	3.85	1.03	1.00				
2.Perceived Work Environment	2.11	0.78	-0.378***	1.00			
3.Perceived Occupational Protection	2.37	0.84	-0.27***	0.43***	1.00		
4.Perceived Welfare Benefits	2.59	0.90	-0.20***	0.42***	0.62***	1.00	
5.Perceived Competency Training	2.43	0.84	-0.09	0.35***	0.50***	0.66***	1.00

*N*=305

^*^P < .10. **P < .05. ***P < .01

### Benchmark regression results

Conducting four times regressions to respectively explore the impact of perceived work environment, occupational protection, welfare benefits, and competency training on turnover intentions of elderly care workers. The results are shown in Table 3. After including personal characteristics and work status control variables, the regression results show that perceived work environment ($\beta$= -0.5164, *p*< 0.01), perceived occupational protection ($\beta$= -0.3390, *p*<0.01), perceived welfare benefits ($\beta$= -0.2620, *p*<0.01), and perceived competency training ($\beta$= -0.1421, *p*<0.1,) of elderly care workers significantly affect turnover intentions. That is to say, the better the perceived human resource management practices of elderly care workers, the lower turnover intentions they have. In addition to this, the results in Table 3 show that the older the elderly care worker is, the higher the turnover intentions they may hold.

**Table 3:** Multiple regression models for turnover intentions (*n*=305)

Variable	(1)	(2)	(3)	(4)
Perceived Work Environment	-0.5164***			
Perceived Occupational Protection		-0.3390***		
Perceived Welfare Benefits			-0.2620***	
Perceived Competency Training				-0.1421*
Gender	0.1140	0.0549	0.0703	0.0540
Age	0.0126**	0.0158***	0.0173***	0.0166***
Education	-0.0474	0.0455	0.0249	-0.0098
Elderly Number	0.1771*	0.1624	0.1765	0.1644
Work Year	0.0806	0.0493	0.0339	0.0102
Certificate	0.0400	-0.0047	0.0460	0.0324
constant	4.1898***	3.6610***	3.4964***	3.3371***
R^2^	0.1937	0.1205	0.0981	0.0619

^*^P < .1, **P < .05, ***P < .01

### Testing for moderating effect of person-job fit

Based on the mean value of person-job fit, we classify samples above the mean value as the “high” group, and samples below the mean value as the “low” group to test the moderating effect of person-job fit. From the results shown in Table 4, each perceived human resource management practice has a significant effect on the turnover intentions of employees with high person-job fit and low person-job fit. After comparing the coefficients, it is found that after considering the person-job fit factor, the perceived work environment (${\upbeta }_{{\text{H}}1}$=-0.4947, ${\upbeta }_{{\text{L}}1}$=-0.5217), perceived occupational protection (${\upbeta }_{{\text{H}}2}$=-0.2675, ${\upbeta }_{{\text{L}}2}$=-0.4739), perceived welfare benefits (${\upbeta }_{{\text{H}}3}$=- 0.2390, ${\upbeta }_{{\text{L}}3}$=-0.2844), and perceived competency training (${\upbeta }_{{\text{H}}4}$=-0.1460, ${\upbeta }_{{\text{L}}4}$=-0.2263) have heterogeneity in relation to turnover intentions, and a high level of person-job fit helps elderly care workers to overcome the negative effects arising from institutional human resource management and reduce their turnover intentions.

**Table 4:** Tests for moderating effects of person-job fit

Variable	Perceived work environment		Perceived occupational protection		Perceived welfare benefits		Perceived competency training	
Group	High	Low	High	Low	High	Low	High	Low
Coefficient	-0.4947***	-0.5217***	-0.2675***	-0.4739***	-0.2390***	-0.2844**	-0.1460*	-0.2263*
SD	0.0935	0.1231	0.0828	0.1137	0.0792	0.1312	0.0840	0.1284
R^2^	0.1932	0.3562	0.1327	0.2687	0.1284	0.1816	0.1022	0.1591

*N* = 226 in High group, *N* = 79 in Low group

^*^P < .1, **P < .05, ***P < .01

### Testing for moderating effect of Institutional ownership

Similarly, based on the mean value of institutional ownership, we classify samples above the mean value as the “high” group, and samples below the mean value as the “low” group to verify the difference between the turnover intentions of elderly care workers in public and private elderly care institutions. Table 5 shows the regression results after grouping and compares the coefficients of the public institution group (Public) with the private institution group (Private). It reveals that the regression coefficients of perceived work environment (${\upbeta }_{{\text{Pub}}1}$=-0.4538, ${\upbeta }_{{\text{Pri}}1}$=-0.6474), perceived occupational protection (${\upbeta }_{{\text{Pub}}2}$=-0.3225, ${\upbeta }_{{\text{Pri}}2}$=-0.4113), and perceived welfare benefits (${\upbeta }_{{\text{Pub}}3}$=-0.2126, ${\upbeta }_{{\text{Pri}}3}$=-0.3574) of elderly care workers all have significantly negative regression coefficients at the 1% level, and the regression coefficients for perceived human resource management practices among elderly care workers in public institutions are greater than in private institutions. The last two columns of the regression results indicate that only the perceived competency training of elderly care workers in private institutions significantly affects the turnover intentions. In conclusion, the public nature of elderly care institutions can buffer the effect of perceived human resource management of elderly care workers on turnover intentions.

**Table 5:** Tests for moderating effects of institutional ownership

Variable	Perceived work environment		Perceived occupational protection		Perceived welfare benefits		Perceived competency training	
Group	Public	Private	Public	Private	Public	Private	Public	Private
Coefficient	-0.4538***	-0.6474***	-0.3225***	-0.4113***	-0.2126***	-0.3574***	-0.0728	-0.2251*
SD	0.0842	0.1381	0.0888	0.1207	0.0805	0.1226	0.0953	0.1160
R^2^	0.1992	0.2274	0.1405	0.1377	0.1037	0.1231	0.0675	0.0874

*N* = 177 in Public group, *N* = 128 in Private group

^*^P < .1, **P < .05, ***P < .01

## Discussion

The aging process of China’s population is deepening, and the shortage of elderly care workers will continue to exist, which means the issue of turnover rate of elderly care workers is gradually receiving attention. Developed countries such as the United States and Singapore have established nursing staff grading systems for Registered Nurse(RN), Licensed Practical Nurse(LVN), and certified Nursing Assistant(NA) [43]. The collaborative team composed of nurses and care workers is efficiently managed, with clear role positioning, and can provide high-quality nursing services [44]. However, there are still serious structural problems in the organizational context of elderly care workers in China at present, such as poor working conditions, weak professional skills, and low certification rate [45]. This study empirically examined how turnover intentions of Chinese elderly care workers was generated from the perspective of perceived human resource management practices. We analyzed the effects of perceived work environment, occupational protection, welfare benefits and competency training on the turnover intentions of 305 elderly care workers from 21 provinces in terms of “perceptions of hygiene” and “perceptions of motivation”, and our findings provided useful insights for researchers and managers.

First, perceived human resource management practices had a negative effect on turnover intentions, and the better the perceived status, the lower the risk of turnover among elderly care workers. The perceived work environment of elderly care workers had the greatest impact on the turnover intentions (${\beta }_{E}$=-0.5164), indicating that the daily work environment of elderly care workers had a deeper impact on the work experience than the occupational protection system (${\beta }_{P}$=-0.3390), the welfare benefits system (${\beta }_{M}$=-0.2620) and the competency training system (${\beta }_{T}$=-0.1421). This is consistent with the findings of some earlier studies, that is, the turnover intentions of elderly care workers arises from the great difference between their ideal role before joining job and the actual working conditions after joining job, and the unsatisfactory working experience of elderly care workers maps out the practical difficulties encountered in the working environment [46]. A global study shows that the nursing shortage caused by high unemployment among nursing staff has affected countries in Europe, Asia and North America, and that working conditions, employment policies and deficiencies in career advancement programs have made the nursing profession less attractive. The reasons behind this phenomenon are diverse. Firstly, the working environment is closely related to the daily work of employees, which is closely related to their work experience. Poor working environment leads to a lack of attractiveness in the elderly care industry [3]; Secondly, the asymmetry between high work intensity and low salary levels makes elderly care workers feel that their work is undervalued and their job content lacks value [47], therefore, many elderly care workers lack work motivation; Thirdly, one important reason why elderly care workers face dual physical and psychological pressures is that they received inadequate training and education, as well as inadequate occupational protection, which are often overlooked by institutions [48]. Elderly care workers is lack of standardized professional skills [49], being misunderstood by elderly people and their families, finally resulting in physical pain and negative work emotions [50]; Finally, as the proportion of young employees in the elderly care workers’ team continues to increase, employees aspire to have reasonable and transparent career promotion channels. They hope that elderly care managers can face the difficulties of nursing work and have their own career promotion paths [13]. If elderly care institutions ignore the promotion needs of employees for a long time, employee may choose to change jobs [20]. In summary, meeting the demand for a good work experience of elderly care workers fundamentally lies in optimizing and improving the internal human resource management system of elderly care institutions from multiple aspects such as work environment, salary and benefits, occupational protection, and skill training.

Second, person-job fit can moderate the relationship between perceived human resource management practices and turnover intentions. In other words, some elderly care workers may give up the idea of turnover when there is a high degree of alignment with the requirements of the job. For example, in the field of hospitality management, person-job fit has been proven to be an important forecast indicators of employee organizational commitment, and the degree of person-job fit mediates employee work sentiment and employee engagement levels [51]. Firstly, employees with high person-job fit usually have good self-efficacy [52]. The higher the match between job skills and job requirements, the more confident individuals are in completing their tasks, believing that their efforts can meet the employer’s requirements and their expectations can be effectively rewarded, so they have positive attitudes toward work [53]. Secondly, a high level of person-job fit is also conducive to the development of strong organizational commitment and leads to employees’ identification with the concept of organizational goals and values [54]. Employees with high person-job fit tend to participate in job design spontaneously and further develop a high level of job responsibility and organizational loyalty, so that they will give up the idea of turnover out of a strong sense of responsibility even when they have a poor work experience. This result emphasizes that elderly institutions should avoid a formalized vocational competency training and assessment and evaluation system, strictly control the staff recruitment and assessment system procedures, and comprehensively promote the labor quality training of staff with the core of improving the operational skills of staff.

Finally, the magnitude of the effect of perceived human resource management practices of elderly care workers on turnover intentions varies by institutional ownership. This finding is similar to most empirical studies that looked at the public and private sectors [55]. A study of 382 nursing staff in the Jordanian health care sector showed that nursing staff working in the public sector perceived their work environment significantly better than nursing staff in the private sector, and that nursing staff in the public sector had higher levels of job well-being [56]. This is because private institutions aim for excessive profits [57] and regulate employees’ work behaviors by setting up a strict performance appraisal system. Employees are prone to burnout and induce turnover intentions; while employees in public institutions spontaneously regulate their psychological tolerance and sense of self-resistance to frustration due to the pursuit of job security [58]. The public interest nature of public institutions creates a buffer between the perceived human resource management practices and the turnover intentions, increasing the tolerance of current employees to a bad work condition and thus reducing the occurrence of impulsive departures. At present, China’s *14th Five-Year Plan for the Development of the National Aging Career and Elderly Care Service System* calls for strengthening the construction of elderly care workers and promoting the reform of elderly care institutions [59]. Under the trend of mainly building public elderly institutions, it has become an inevitable requirement to build an inclusive elderly service system and promote the healthy development of the aging career by paying attention to the working condition needs of elderly care workers in public elderly institutions and reducing occupational mobility.

The study has some limitations. First, this study only focused on the role of perceived human resource practices of elderly care workers on turnover intentions, and other possible environmental factors such as organizational equity and organizational culture could be further considered in future studies. Second, this study was not hierarchically divided according to the differences in the individual characteristics of the study participants, and the next study could focus on specific levels of the elderly care workers to explore in order to further refine the findings.

## Conclusion

For a long time, China’s elderly care service industry has been facing the dilemma of talent shortage. This study shows that elderly care workers may develop the idea of turnover when they perceive low level of human resource management in the institution, and this idea may change because of the differences in work ability and institutional ownership. The conclusion suggests that managers of elderly care institutions should develop recruitment criteria for elderly care workers based on the institutional ownership structure, pay attention to the working environment, welfare and benefits, competency training of employees, and focus on occupational protection for elderly care workers . This paper is a complement to previous research on the factors influencing turnover intentions of elderly care workers. For a long time, China’s elderly care service industry has been facing the dilemma of talent shortage. In the environment of “public-run and private-sector-supplemented” elderly care institutions in China, on the one hand, elderly care institutions should review the shortcomings of human resource management, optimize internal management mechanisms, provide stable and good working conditions for elderly care workers, and motivate their enthusiasm to work; on the other hand, the government and elderly care institutions should work to develop and improve professional standards and job requirements for elderly care service, and to ensure that carers for the elderly receive adequate knowledge education and competency training before they are formally hired. Finally, they should promote the public welfare value of elderly care services, encourage elderly care workers to conclude organizational commitments, and strengthen their sense of honor and mission, so as to better solve the dilemma of turnover of elderly care workers.

### Supplementary Information

**Supplementary Material 1.**

## Data Availability

All the data used during the study are available from the corresponding author on reasonable request.

## Abbreviation

HRM: Human Resource Management

## Footnotes

1 The ranking of provinces’ population aging degree is based on the proportion of the elderly aged over 60 in each province according to the data of the Seventh National Population Census.

2 The questionnaire was personal developed for this study and have not been published elsewhere.
