# Supplementary material for: Perceived HRM and turnover intentions of elderly care workers: perspective from person-job fit and institutional ownership
Source: BMC Nurs. 2024 Apr 15;23:242. doi: 10.1186/s12912-024-01926-9 (PMC11020918; doi:10.1186/s12912-024-01926-9)
Supplement: Supplementary file 1 — Supplementary Material 1. [file 12912_2024_1926_MOESM1_ESM.pdf]

Research questionnaire on the construction of elderly care worker team in China

Hello! We are members of the research group on the construction of China's elderly care worker team. We hope to understand the current situation of the construction of China's elderly care service worker team through questionnaires, and provide policy suggestions for achieving the professionalization and systematization of the elderly care worker team. This survey questionnaire is anonymous and targets elderly care workers engaged in elderly care service institutions. The questionnaire is only for scientific research projects, and we will strictly keep your information confidential. Answering the question may take up about 10 minutes for you. Please read the question and fill out the questionnaire truthfully. Thank you for your participation and cooperation!

Individual characteristics

1. What is your gender?

① male

② female

2. How old are you?

____

3. what's your education level?

① primary school and below

② junior high school, junior college or high school

③ college or university undergraduate

④ master's degree and above

4. What province are you work in?

____

5. How is your previous learning experience relevant to your current job?

① very relevant

② relatively relevant

③ average

④ not very relevant

⑤ very irrelevant

6. What is your current level of understanding about the elderly care worker team?

① very familiar

② relatively familiar

③ average

④ not very familiar

⑤ very unfamiliar

7. Do you think your college’s job skills can meet the job requirements?

① very sufficient

② relatively sufficient

③ average

④ not very sufficient

⑤ very insufficient

Work status

8. Have you undergone systematic training before getting this job?

① yes

② no

9. Have you obtained any professional qualification certificate?

① yes

② no

10. How many elderly people do you serve every day?

① 0-10 people

② 11-20 people

③ 21-30 people

11. How many years of work experience do you have?

① 5 years or less

② (5, 10] years

③ (10, 15] years

④ (15, 20] years

⑤ more than 20 years

12. How much is your current monthly income approximately?

① (1000, 3000] yuan

② (3000, 5000] yuan

③ (5000, 7000] yuan

④ (7000, 9000] yuan

⑤ more than 9000 yuan

13. How do you think the current work burden?

① very high

② relatively high

③ average

④ not very high

⑤ very low

Person-job fit and institutional ownership

14. How relevant is your previous study or work experience to your current job?

① very relevant

② relatively relevant

③ average

④ not very relevant

⑤ very irrelevant

15. What is the nature of your workplace?

① public institution

② private institution

Turnover intention and Perceived HRM

16. Do you think the current working environment of the institution is perfect?

① very satisfied

② fairly satisfied

③ average

④ not very satisfied

⑤ very dissatisfied

17. Do you think the current occupational protection of the institution is perfect?

① very satisfied

② fairly satisfied

③ average

④ not very satisfied

⑤ very dissatisfied

18. Do you think the current welfare benefits of the institution is perfect?

① very satisfied

② fairly satisfied

③ average

④ not very satisfied

⑤ very dissatisfied

19. Do you think the current competency training of the institution is perfect?

① very satisfied

② fairly satisfied

③ average

④ not very satisfied

⑤ very dissatisfied

20. How willing are you to turnover at this time?

① very strong

② relatively strong

③ average

④ occasionally want to turnover

⑤ will not turnover

21. Are you still willing to work in elderly care services in the future?

① very willing

② relatively willing

③ average

④ Not very willing

⑤ Not willing
